# Diverse Superatomic Magnetic and Spin Properties of Au_144_(SC_8_H_9_)_60_ Clusters

**DOI:** 10.1021/acscentsci.5c00139

**Published:** 2025-05-29

**Authors:** Juniper Foxley, Marcus Tofanelli, Jane A. Knappenberger, Christopher J. Ackerson, Kenneth L. Knappenberger

**Affiliations:** † Department of Chemistry, 8082 Pennsylvania State University, University Park, Pennsylvania 16802, United States; ‡ Department of Chemistry, 3447 Colorado State University, Fort Collins, Colorado 80523, United States; § Department of Physics, 8082 Pennsylvania State University, University Park, Pennsylvania 16802, United States

## Abstract

Au_144_(SC_8_H_9_)_60_, a colloidal cluster with a 1.7 nm inorganic diameter, exhibits both metallic and molecular-like behavior, along with a distribution of unfilled superatom states. Its 1.7–2.5 eV electronic transitions were probed with variable-temperature, variable-field magnetic circular dichroism (VTV $\overset{⇀}{H}$ -MCD), revealing two energy regions with distinct responses. Below 2.0 eV, MCD transitions exhibited diverse VTV $\overset{⇀}{H}$ responses, including both paramagnetic and diamagnetic behavior, implicating multiple nondegenerate initial states originating within the open-shell superatom S, D, and H HOMO manifold. Above 2.0 eV, uniform field-dependent responses suggested spin-vibronic coupling due to metal–ligand mixing. The Au_144_(SC_8_H_9_)_60_ magneto-optical response is surprisingly complex given the system’s high electronic-state density; discrete structural domains of the cluster, including the superatomic metal core, likely contribute to this diversity. These results show the potential to investigate and tailor the magneto-optical and spin properties of these clusters through structurally precise synthesis and also identify superatomic colloids as candidates for advancing spin-based technologies.

## Graphical abstract

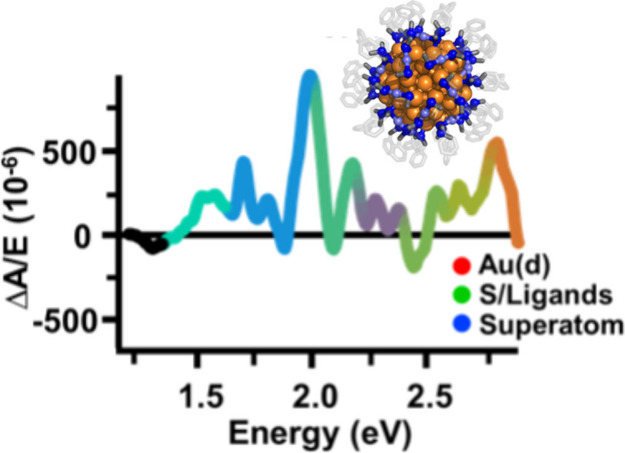

## Introduction

Gold nanostructures have been studied extensively because of their potential use in photonics, sensing, therapeutics, and catalysis applications.^1–8^ A less-explored but equally promising research area involves the magnetic and spin-dependent properties of gold nanoparticles. Electronic spin plays a key role in chemistry by determining the outcomes of many important catalytic processes, including enzyme-mediated reactions, the reduction of CO_2_, and surface-driven molecular reactions.^9–12^ Spin is also an essential component of quantum information sciences. Many examples of high-performance quantum computation rely on trapped atomic ions.^13,14^ However, translation of these gas-phase concepts to scalable condensed-phase devices often results in computational error due to shortened spin lifetimes and large spin-environment interactions.^15^ Superatomic metal clusters, which exhibit atom-like electronic valence structure,^16^ provide an opportunity to combine the spin properties of atoms with the scalable synthesis of catalytic colloids.

The origins of spin-dependent properties in metals depend on nanostructure size, morphology, and composition.^17^ In the case of subnanometer gold, electronic valence plays a determining role in nanocluster magnetism. For example, electrochemical oxidation of anionic Au_25_(SC_8_H_9_)_18_^–^ to a neutral species creates a seven-electron open-shell superatomic system that is paramagnetic, as confirmed by electron paramagnetic resonance (EPR).^18^ Owing to large spin–orbit coupling in gold clusters,^19,20^ photoexcitation of these systems leads to spin-polarized emission, which can be tuned by metal-atom substitution.^21,22^ For slightly larger clusters, such as Au_102_(pMBA)_44_, structure-dependent magnetic properties arise due to thermal deformations in the metal kernel.^23^ By comparison, the optical properties of gold nanoparticles with more than 459 metal atoms are dominated by surface plasmon resonances (SPRs) and exhibit magneto-absorption lineshapes that are determined by nanoparticle morphology.^24,25^ For nanorods, increases in the length-to-diameter aspect ratio transform the SPR magneto-absorption spectrum from a bisignate to a Gaussian line shape.^26^ Hence, even over a relatively small size range, gold nanoparticles exhibit expansive magneto-optical behaviors that can be tailored synthetically.

Although the ultrasmall nonmetallic and plasmon-supporting metallic regimes are both reasonably well understood, little is known about the magnetic and spin properties of gold particles on the cusp of metallicity, which are capable of supporting superatom states of high angular momentum. Monolayer-protected clusters (MPCs) are an ideal platform for understanding gold properties at this intersection because they can be synthesized and isolated with both compositional and size control at the atomic level,^27,28^ enabling correlation between magneto-optical properties and structural influences. Au_144_(SR)_60_ clusters (Figure 1), which exhibit both molecular and bulk-like metallic properties^29^ are an ideal MPC for studying nanoscale metals at the onset of metallicity. Au_144_(SR)_60_ consists of a 114-gold-atom “grand core,” composed of a core–shell–shell structure (Figure 1a). The grand core, which has a diameter of ∼1.7 nm, is protected by 30 (RS-Au­(I)-SR) semiring units (Figures 1b,c), where R is a terminating phenylethanethiol (Figure 1d). The Au_144_(SR)_60_ MPC follows the double-layer capacitance model of electrochemical charging (Figure S1), indicative of a metallic particle.^30^ Following 400 nm excitation, the electron–phonon coupling rates also follow two-temperature model predictions for a Fermi gas of a metallic particle.^31,32^ Taken together, the electrochemical charging and electronic relaxation rates suggest metallic behavior by Au_144_(SR)_60_. However, evidence of single-electron transitions, typical of nonmetallic domains, is also observed in the electronic excitation spectrum^33^ and low-temperature absorption data (Figure S2). In addition, Au_144_(SC_8_H_9_)_60_ has previously been shown to exhibit magneto-optical properties consistent with many individual electronic excitations spanning the visible absorption region.^34^

**Figure 1:**
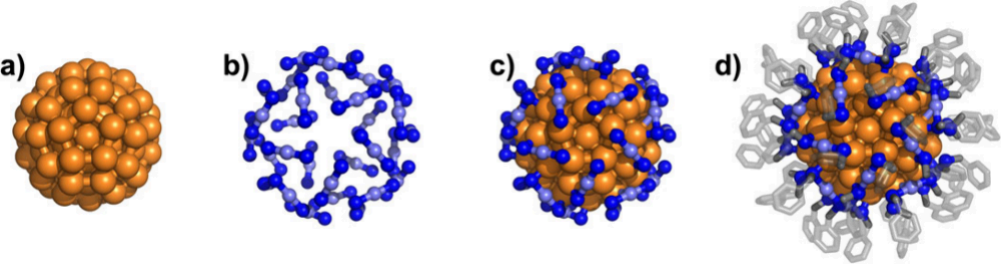
Structure of Au_144_(SC_8_H_9_)_60_. The cluster consists of three structural components: 1) an icosahedral Au_114_ core, shown in a), which is surrounded by 2) 30 S-Au-S semiring units, shown in b) and c), and 3) 60 passivating organic phenyl­ethane­thiol ligands, shown in d).

Density functional theory (DFT) calculation results indicate the complex Au_144_(SC_8_H_9_)_60_ absorption behavior is due, in part, to energy-dependent changes in the relative contributions of states associated with different structural components.^35,36^ This includes contributions from the superatomic orbitals that originate from Au(0) atoms within the cluster’s core. The superatom assignment of Au_144_(SC_8_H_9_)_60_ involves a total of 84 electrons in an open-shell orbital structure, falling short of the 92 electrons that would be required for shell closing.^35^ This results in a HOMO–LUMO manifold comprising a mixture of unfilled 2D, 3S, and 1H orbitals, all of which can impart paramagnetic character to the cluster and influence spin properties.

Variable-temperature, variable-field magnetic circular dichroism (VTV $\overset{⇀}{H}$ -MCD) experiments allow assignment of the spectroscopic transitions of a sample, including magnetic, electronic, and spin properties. In the presence of an applied magnetic field, electronic states undergo Zeeman splitting (Figure 2), and the effects of the magnetic field on excited states, mixing between states, and ground states, respectively, are described by the Faraday A-, B-, and C-terms.^37^ The overall MCD signal, ΔA/E, is related to these three terms according to the formalism

$$\frac{\Delta A}{E}=\gamma \mu_{B}\overset{⇀}{H}\left[\left(A\frac{\partial f\left(E\right)}{\partial E}\right)+\left(B+\frac{C}{k_{B}T}\right)f\left(E\right)\right]$$ 1

where E is energy, f­(E) is a Gaussian lineshape function, γ is the gyromagnetic ratio, μ_*B*_ is the Bohr magneton, $\overset{⇀}{H}$ is the applied magnetic field strength, *k*_*B*_ is the Boltzmann constant, and T is the sample temperature. MCD intensity is defined as the difference between the left- and right-circularly polarized absorption. The B and C terms are both characterized by Gaussian spectral profiles. The B term, which is characteristic of transitions with many near-degenerate states, displays a linear dependence of MCD intensity on magnetic field strength. However, the C term, which dominates for paramagnetic species at low temperatures, can be distinguished by having both a temperature-dependent magneto-optical response as well as saturation of MCD intensity at high magnetic field strengths.^38^ These behaviors arise due to the differences in the relative populations of Zeeman-split states when the applied field strength causes energy differences between electronic states that exceed the thermal energy of the sample.

**Figure 2:**
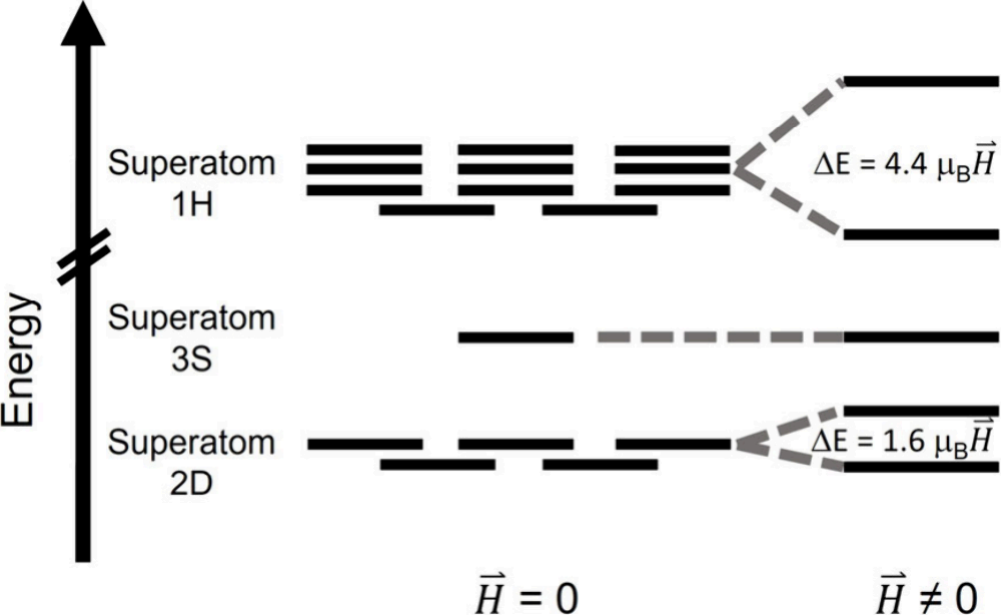
Qualitative illustration of the Zeeman effect on splitting of the HOMO–LUMO manifold of Au_144_(SC_8_H_9_)_60_. For the purposes of illustration, distinct angular momenta states within the 2D, 3S, and 1H orbitals are assumed to be nearly degenerate at zero field. For simplicity, the splitting of only one D and H state in the presence of a magnetic field is shown.

Because Au_144_(SC_8_H_9_)_60_ displays properties consistent with both metallic and nonmetallic behavior, it is an important system for understanding the electronic and magnetic properties of quantum-confined metals approaching classical length scales. This manuscript describes the use of MCD to understand the electronic transitions of Au_144_(SC_8_H_9_)_60_ and examines the excitation energy-dependent effects on its electronic properties, thus improving understanding of how nanoscale structure influences the optical, electronic, and spin properties of large clusters approaching classical metallicity.

## Results and Discussion

MCD spectra spanning 1.3 to 2.7 eV, shown in Figure 3, were acquired at several applied magnetic field strengths (2–10 T; Figure 3a) and temperatures (1.6–90 K; Figure 3b). At 1.6 K, 19 individual peaks were distinguished across the MCD spectrum—a departure from expectations for a classical, metallic nanoparticle. The qualitative $\overset{⇀}{H}$ -dependent response of the spectrum was transition specific, with most peaks presenting unique responses to the applied magnetic field. Within the detected spectral region, even qualitative inspection of the Figure 3a data revealed that both saturating and nonsaturating V $\overset{⇀}{H}$ -MCD peaks coexist within close energetic proximity. For example, the amplitude of the ∼1.7 eV peak saturates at a magnetic field strength of 6 T, whereas the amplitude of the 2.0 eV peak continues to increase at higher magnetic field strengths. These differences highlight the transition specificity of this cluster’s field-dependent MCD responses.

**Figure 3:**
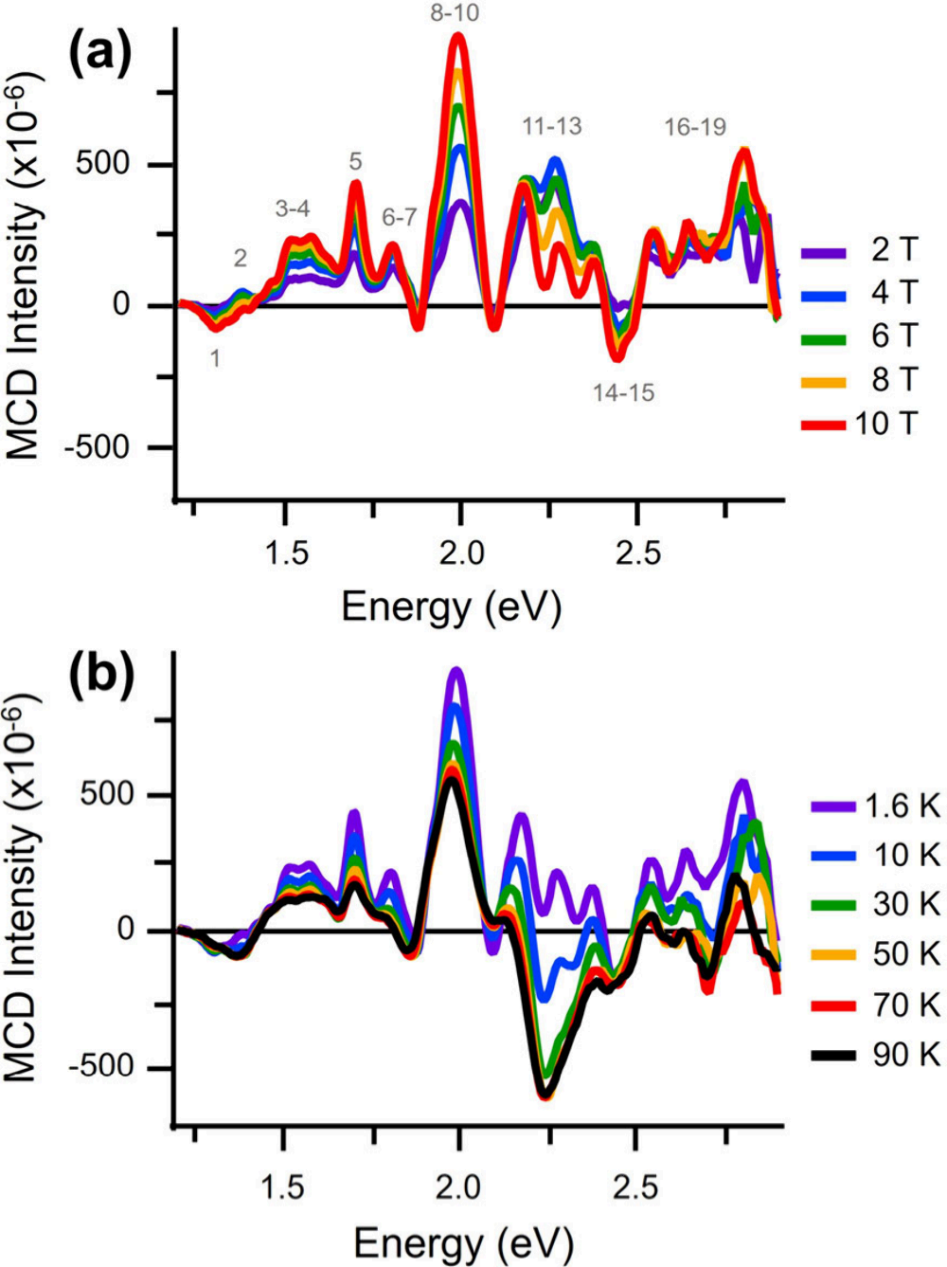
MCD spectra of Au_144_(SC_8_H_9_)_60_. Nineteen resolvable features were detected in the range spanning 1.4–3.0 eV. Peak characteristics are given in Table 1 and Table S1. (a) 1.6 K spectra acquired at several magnetic field strengths up to 10 T, demonstrating evidence of both saturating and nonsaturating field dependencies. (b) 10 T spectra acquired at sample temperatures ranging from 1.6 to 90 K. Amplitudes of most resolvable features decrease with increasing temperature. At temperatures >10 K, a strong negative-polarity feature dominates the spectral region between 2.1 and 2.5 eV.

The temperature response of the cluster was similarly complex. Although the majority of MCD signals exhibited a decrease in amplitude when sample temperature was increased (indicating significant C-term contributions to the MCD spectrum), a deviation from this pattern is observed between 2.1 and 2.5 eV. At low temperatures, this region displays three distinguishable positive-polarity MCD peaks at 2.2, 2.3, and 2.4 eV, respectively (peaks 11–13). The typical expectation for C-term-based transitions is an inverse dependence of signal amplitude on sample temperature. However, at sample temperatures at or above 10 K, the MCD spectra were no longer characterized by three prominent and distinct peaks in the 2.0–2.5 eV region; rather, these peaks gave way to a broad, featureless negative-polarity component, causing an inversion of the net MCD polarity. Owing to this complexity, the field and temperature dependencies of the MCD spectra for Au_144_(SC_8_H_9_)_60_ will be discussed in terms of two spectral regions, which correspond to excitation energies below and above 2.0 eV.

### I. Region 1: 1.7 to 2.0 eV

The VTV $\overset{⇀}{H}$ -MCD response of spectral region 1 is summarized in Figure S4 and Figure 3. Figure S4a contains an overlay of V $\overset{⇀}{H}$ -MCD spectra acquired at 2–10 T and sample temperature of 1.6 K; five different peaks were resolved in the 300 meV-wide range between 1.7 and 2.0 eV. Those at 1.7 and 1.8 eV show apparent amplitude saturation at high applied magnetic field strengths, suggestive of paramagnetic centers in Au_144_(SC_8_H_9_)_60_. By comparison, amplitudes for the other peaks continue to increase at higher field strengths. To examine these transitions further, MCD data were obtained at 10 T for a variety of sample temperatures (Figure S4b). For four of the five transitions, the magnitude of the MCD signal decreases as the sample temperature increases, reflecting paramagnetic behavior and nonzero Faraday C-term contributions. The one exception is the peak at 1.85 eV; for this peak, the MCD amplitude remained consistent despite changes in sample temperature, suggesting either minimal Faraday C-term or substantial B-term contributions.

The magnetic field-dependent MCD intensity of four selected peaks (Peak 5, ∼1.7 eV: Figure 4a; Peak 6, 1.8 eV: Figure 4b; Peak 7, 1.85 eV: Figure 4c; and Peak 8, 1.9 eV: Figure 4d) are shown in Figure 4. For the 1.8 eV peak, a saturation of MCD intensity at high magnetic field strengths (approximately 6 T) was observed with qualitatively low linear (i.e., B-term) contribution, suggesting a paramagnetic center with a small degree of mixing by near-degenerate states.^28^ In contrast, the spectral feature at 1.85 eV (Peak 7) displays a linear MCD intensity dependence with respect to applied field strengths (Figure 4c). The dominance of the Faraday B-term for this peak reflects a diamagnetic response and indicates extensive field-induced mixing of several near-degenerate states.^38^ Other peaks within this region, such as those at 1.7 eV (Peak 5) and 1.9 eV (Peak 8) (Figures 4a,d, respectively), are characterized by a sum of contributions from both C- and B-term components. The variety of responses reflects the existence of a complex manifold of electronic states that serve as the initial states for distinct MCD transitions. This diversity of MCD magnetization responses is attributed to the differences in angular momenta of core-based HOMO–LUMO states of Au_144_(SC_8_H_9_)_60_.

**Figure 4:**
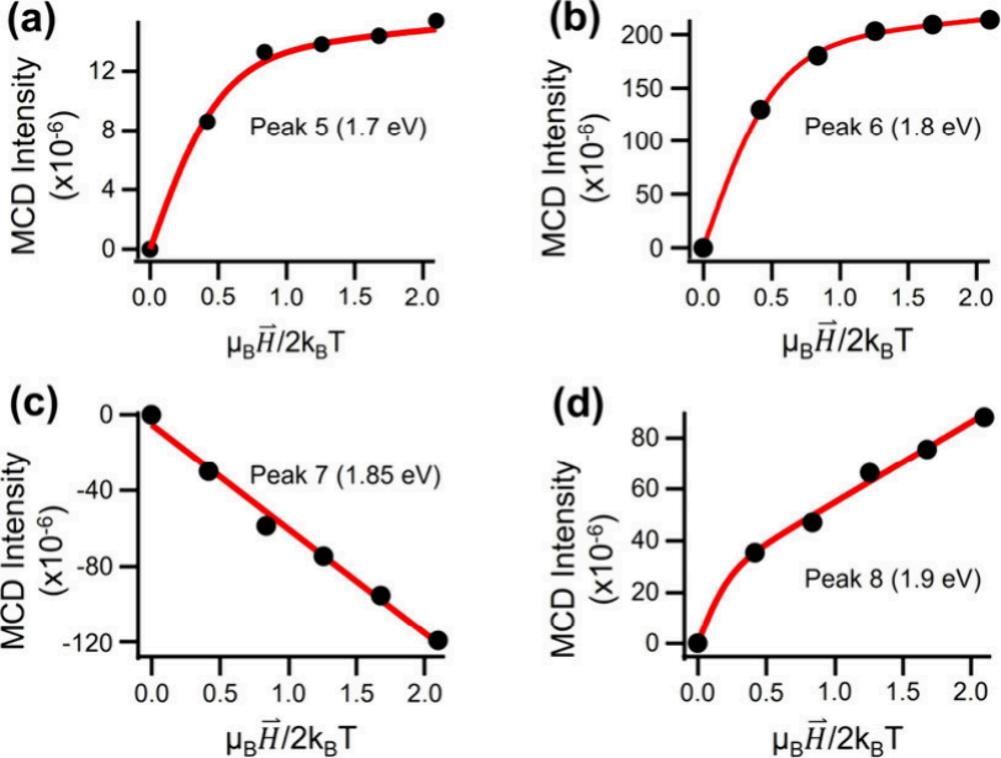
Variable-temperature, variable magnetic field-dependent responses of select Au_144_(SC_8_H_9_)_60_ MCD peaks within the range from 1.7 to 2.1 eV. Red lines correspond to fits of the MCD data to eq 2. (a) A saturating MCD component dominates the response of the 1.7 eV transition (Peak 6), suggesting significant paramagnetic character. The positive-amplitude linear component results from a B-term contribution, which reflects mixing of the paramagnetic transition with another state. (b) The VTV $\overset{⇀}{H}$ -MCD response acquired at 1.8 eV (Peak 6) exhibits complete saturation, consistent with excitation of a paramagnetic center. (c) The linear VTV $\overset{⇀}{H}$ -MCD response of the 1.85 eV peak (Peak 7) reflects the diamagnetic nature of this transition. (d) VTV $\overset{⇀}{H}$ -MCD response acquired at 1.9 eV (Peak 8). This feature displays both saturating and linear contributions, and data fitting yields a distinct Landé g value of 4.4 ± 1.1, indicating a large angular momentum for the ground state.

To analyze the relative contributions of C-term and B-term components to the MCD signals quantitatively, the VTV $\overset{⇀}{H}$ magnetization curves shown in Figure 4 were fit to

$$\frac{\Delta A}{E}=C\;\tanh\left(\frac{\mu_{B}\overset{⇀}{H}g}{2k_{B}T}\right)+B\overset{⇀}{H}$$ 2

where B and C are the relative amplitude coefficients of Faraday B- and C-term contributions, respectively,^38,39^ and g is the transition-specific Landé g-factor, which accounts for both the spin and orbital components of the total angular momentum (see Figure S5 and supplemental discussion). Deviations from the free-electron g-factor (g = 2.0023) are a consequence of state-specific spin–orbit coupling for quantum-confined gold. For the MCD peaks at 1.7, 1.8 and 1.9 eV (Peak 5, 6, and 8, respectively), fitting to eq 2 yielded Landé g values of 1.9 ± 0.3, 1.6 ± 0.3 and 4.4 ± 1.1, respectively. The g-factors and percentage of Faraday B-terms for each peak are reported in Table 1. The large g-factor obtained for Peak 8 (1.9 eV) may suggest an initial state with a very high orbital or spin (or both) angular momentum, which would cause greater susceptibility to the field-induced Zeeman splitting. This is contrasted with the comparatively low Landé g values obtained for peaks 5 and 6 (1.7 and 1.8 eV).

**Table 1:** Landé g and Percent B-Term Contributions for MCD Spectral Features Determined by Fitting Magnetization Curves to Eq 2

Peak Number	Peak Energy (eV)	Lande g-Factor	% B Term
5	1.7	1.9 ± 0.3	30
6	1.8	1.6 ± 0.3	<10
7	1.85	2.0 ± 0.8	100
8	1.9	4.4 ± 1.1	60
9	2.0	2.1 ± 0.5	45
10	2.1	--	--
11	2.2	2.3 ± 0.3	<10
12	2.3	1.9 ± 0.3	25
13	2.4	2.1 ± 0.4	20
14	2.45	--	100

To understand better the origin of the different Au_144_(SC_8_H_9_)_60_ magneto-optical responses, the MCD spectra were correlated with previously published DFT calculations. The results of this comparison are shown in Figure 5, which contains RGB color values that were assigned based on the relative spectral contributions of each structural domain.^40^ These calculations indicate that orbital contributions to the electronic excitation of Au_144_(SR)_60_ arise from different structural domains, with lower-energy (1.4–1.6 eV) excitations largely dominated by ligand-centered transitions and higher-energy contributions mostly arising from metal-centered transitions.^40^ The predicted relative contributions of different structural components to the electronic excitation are reflected by the color coding of the MCD spectrum in Figure 5. Metal–ligand transitions (S/Ligand) are represented by green, metal-centered (Au­(d)) are red, and superatom contributions are blue. The region resolved within these experiments (1.5–2.8 eV) largely represented metal–ligand transitions; metal-centered excitations predominantly occur at energies ≥2.8 eV. These two components are overwhelmed in the range from 1.7 to 1.9 eV, where the relative contribution of superatomic orbitals reaches a maximum and becomes the majority component. This coincides with the spectral region where both the largest diversity of magneto-optical responses was observed and paramagnetic transitions dominated the MCD response, indicating that unfilled cluster superatom states are responsible for the paramagnetic character of Au_144_(SC_8_H_9_)_60_ reported here. Hence, MPC superatoms provide opportunities to observe unique magneto-optical phenomena in nanoscale metals.

**Figure 5:**
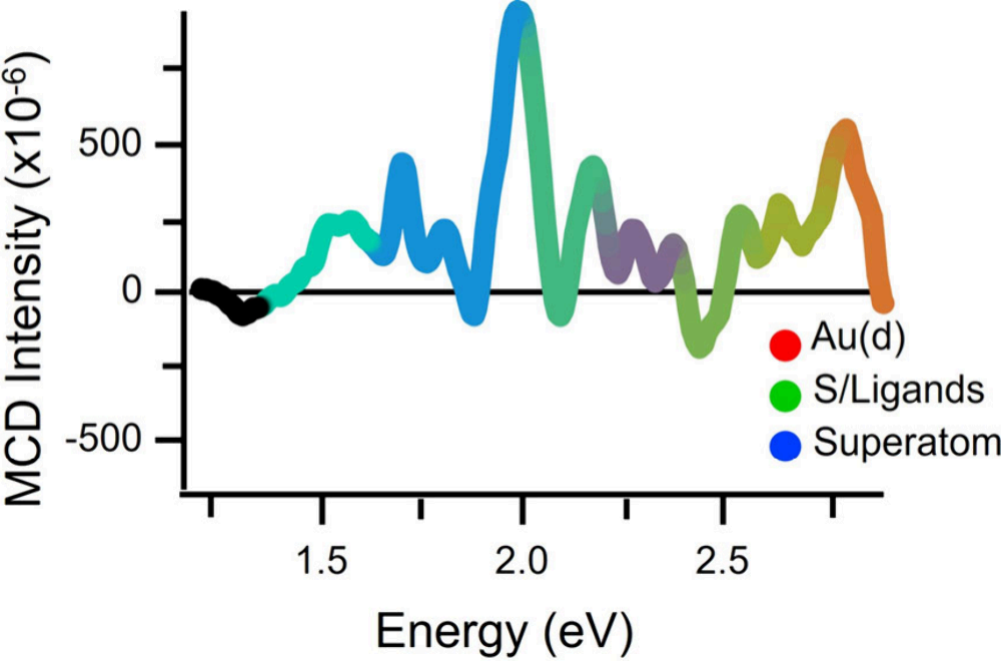
MCD spectrum of Au_144_(SC_8_H_9_)_60_ at 1.6 K and 10 T color-coded according to expected electronic-state contributions to the excitation from previously published DFT calculations. RGB color values are assigned based on the percent contributions of Au­(d) (red), S/Ligands (green), and superatom (blue) proportions.

The paramagnetic behavior observed at both 1.8 and 1.9 eV in Figures 4b,d, respectively, suggests these features arise from within the unfilled superatom HOMO manifold, albeit from different orbitals within that manifold. For peak 6 (1.8 eV), a small B-term contribution implies only minor field-induced mixing with near-degenerate states. Rather, the observed intensity at increasing magnetic field indicates a significant Faraday C-term contribution. The apparent observed Landé g value of 1.6 ± 0.3 for peak 6 makes precise assignment of the initial state difficult. However, theoretical assignments of orbitals within the HOMO–LUMO region of Au_144_(SR)_60_ clusters^35,36^ combined with the observed paramagnetic character and energetic isolation of the initial state suggest that the transition involved excitation from the 2D superatom orbital. The most consistent assignment for this feature would be either ^4^D_3/2_ or ^4^D_5/2_. Peak 8 (1.9 eV) is assigned to either ^12^H_1/2_ (g = 5.3) or ^6^D_1/2_ (g = 3.3) origins; the strong linear component for its field dependency supports this assignment because splitting of the H orbital under icosahedral symmetry produces a manifold of near-degenerate states. For peak 7 (1.85 eV), the linear dependence of MCD intensity on applied magnetic field strength implicates excitation originating from mixed states. In combination with the lack of an apparent Faraday C-term contribution, this transition is attributed to excitation from filled-shell orbitals below the HOMO. Theoretical results have previously shown that these orbitals display mixed character, supporting this assignment.^40^ Hence, while these features all present paramagnetic character, each arises from a distinct initial state. The large range of responses highlights a benefit of superatomic gold clusters—a variety of electronic orbital, spin, and magnetic properties can be accessed for a single nanocluster.

### II. Region 2: 2.0 to 2.5 eV

Figure 6a compares the field-dependent MCD spectra of Au_144_(SC_8_H_9_)_60_ in the region between 2.0 and 2.5 eV (Peaks 10–14) acquired at a sample temperature of 1.6 K. Within this energy range, the MCD spectra were dominated by three peaks centered at 2.2, 2.3, and 2.4 eV. The corresponding magnetization curves are shown in Figure 6b (Peak 11, 2.2 eV), Figure 6c (Peak 12, 2.3 eV), and Figure 6d (Peak 13, 2.4 eV). The magnetization isotherms for each of these MCD peaks display both a majority positive-amplitude C-term component and a negative-polarity B-term component. When fit to eq 2, the magnetization curves result in Landé g values of 2.3 ± 0.4 (Peak 11, 2.2 eV), 1.9 ± 0.3 (Peak 12, 2.3 eV), and 2.1 ± 0.4 (Peak 13, 2.4 eV). The similarity of the Landé g-factor values, which were within error of each other, reflect related responses to the applied magnetic field, suggesting common origins for these transitions. DFT calculations predict that sulfur semiring ligands are the main contributors to excitation within this region.^35^ As such, these peaks may correspond to metal–ligand excitation from sulfur p orbitals within the gold–sulfur semiring ligand band. Further evidence of ligand-centered MCD responses is observed in the temperature-dependent MCD response. The 10 T MCD spectra measured between 2.0 and 2.5 eV and acquired at several sample temperatures are plotted in Figure S6. The presence of regular energy splitting between the three positive features (peaks 11, 12, and 13) is highlighted in Figure 6a and the inset of Figure S6a, where the MCD spectrum taken at 10 T and 1.6 K is isolated. These features display a regular spacing of approximately 100 ± 15 meV (800 ± 125 cm^–1^), which may correspond to a spin-vibronic progression in this region. Previously published vibrational analysis of Au_144_(SC_8_H_9_)_60_ suggests that this energy spacing agrees well with normal modes of the phenylethanethiol ligand,^41^ implicating a mixing of organic ligand modes with electronic excitations centered within the sulfur p orbitals and further supporting the assignment of excitations within this region to the ligand band. Also consistent with this assignment are the larger Faraday B-term contributions, particularly those noted for peaks 12 and 13 (Table 1). Because a vibronic transition involves nonadiabatic coupling between at least two states, the observation of field-induced mixing is consistent with expectations for this type of transition. Further evidence for combined C- and B-term contributions comes from the complex temperature dependence of the MCD response, which displays a distinct inversion of polarity with increasing temperature (Figures S6a,b). At 1.6 K, the spectrum was dominated by C-term contributions, which generally dominate the low-temperature MCD response of paramagnetic systems;^42,43^ these gave way to B-term signals as the sample temperature was increased. Taken together, the magnetization curves (Figure 6) and the regular peak spacing of the MCD signals (Figure 6a and Figure S6a) are consistent with nonadiabatic spin-vibronic interactions.

**Figure 6:**
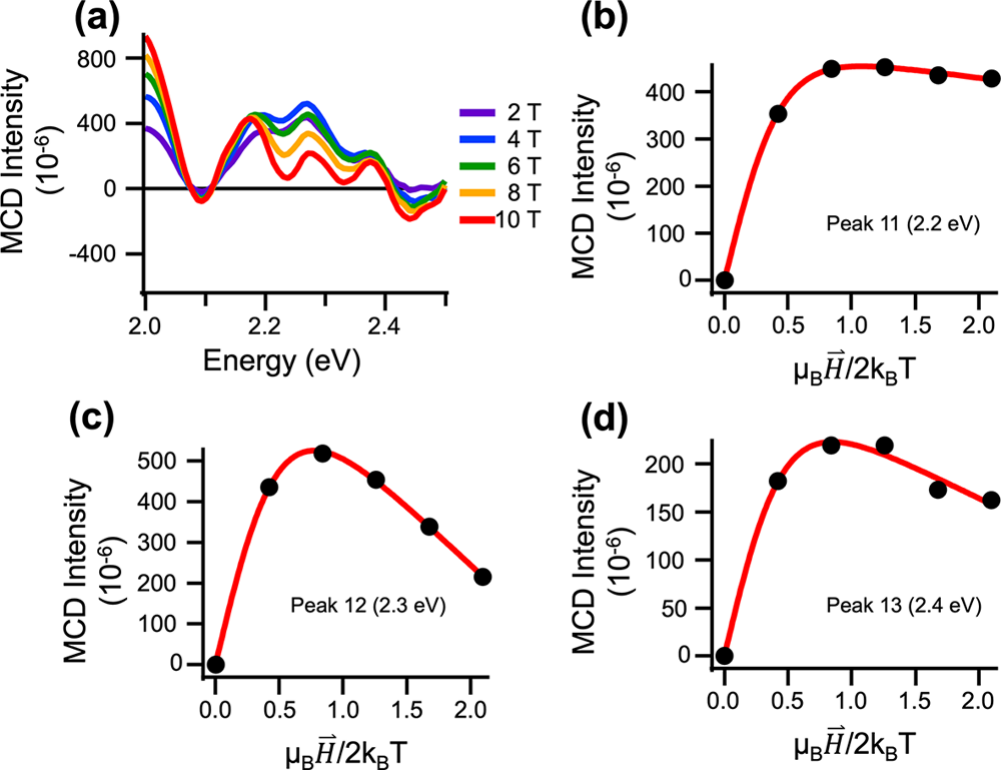
MCD spectra and responses of Au_144_(SC_8_H_9_)_60_ measured in the range of 2.0 to 2.5 eV. (a) Overlay of MCD spectra acquired at several magnetic field strengths, spanning 2–10 T and a sample temperature of 1.6 K. Variable-temperature, variable magnetic field response for peaks centered at 2.2 eV (b), 2.3 eV (c), and 2.4 eV (d). Red lines correspond to fits of the MCD data (black circles) to eq 2.

## Conclusions

Here, the surprisingly complex magneto-optical properties of Au_144_(SC_8_H_9_)_60_ were described. In contrast to expectations for a metallic nanoparticle, the MCD spectra of Au_144_(SC_8_H_9_)_60_ exhibited 19 distinct peaks that showed a diverse range of magnetic-field dependencies. Between 1.7 and 2.0 eV, the observed MCD responses reflect a variety of electronic properties including paramagnetic, diamagnetic, heavily mixed, and high-angular-momenta transitions. In particular, paramagnetism is attributed to unpaired electrons in a manifold of partially filled superatom states. The superatom character of condensed-phase monolayer-protected clusters is a unique feature of these colloidal nanoparticles, which endows electronic, spin, and magnetic properties that are not easily achievable in other colloidal materials. The high angular momenta associated with these states, including the identification of superatom H orbitals, may provide new opportunities to explore spin-dependent chemistry. In particular, electronic spin may be used to catalyze new and more efficient reactions, especially those involving multistep and multielectron processes. Superatoms may also provide a bridge between gas-phase atom concepts and scalable components of quantum computers. Between 2.1 and 2.5 eV, the MCD transitions originate from primarily metal–ligand band contributions. As such, structural control over the metal–ligand interface may enable synthetic approaches to controlling nanocluster magnetism and spin properties. The excitation-dependent MCD properties of Au_144_(SC_8_H_9_)_60_ provide insight into the complex magnetic, spin, and electronic properties of gold colloids on the cusp of classical metallicity. The results suggest that structurally precise superatomic gold clusters may provide novel chemistry solutions for spin-based technologies.

## Methods

Au_144_(SC_8_H_9_)_60_ MPCs were synthesized according to previously reported protocols.^32^ Solutions of MPCs were prepared in a 10% polystyrene in toluene solution. Sample solutions were then dropcast onto a quartz coverslip, covered by a second coverslip, and dried in a vacuum desiccator. Prepared slides were adhered to the cryostat probe via copper tape. The sample and probe were then inserted into the sample chamber of a ^3^He-cooled superconducting 10 T magnet (Oxford). Sample temperature was modulated via resistive heating. Absorption and MCD measurements employed a quartz-tungsten-halogen (QTH) 250 W lamp (Newport) broadband source focused onto the sample. Polarization of light at the sample was adjusted through use of a photoelastic modulator. Following interaction with the sample, a diffraction grating scanned the spectrum across a Si photodiode for collection of spectra. Differential absorption MCD spectra were calculated using data collected at positive and negative field polarities, which were accomplished through inversion of the circular polarization of the incident light onto the sample. A lock-in amplifier was used to synchronize absorption collection to polarization states.

## Supplementary Material





## Notes

Au_144_(SC_8_H_9_)_60_ synthesis and characterization details, experimental spectra, and all MCD peaks observed (PDF)
Transparent Peer Review report available (PDF)
