# Supplementary material for: Diverse Superatomic Magnetic and Spin Properties of Au144(SC8H9)60 Clusters
Source: ACS Cent Sci. 2025 May 29;11(8):1329–35. doi: 10.1021/acscentsci.5c00139 (PMC12395290; doi:10.1021/acscentsci.5c00139)
Supplement: Supplementary file 1 [file oc5c00139_si_001.pdf]

Supporting Information for:

Diverse Superatomic Magnetic and Spin Properties of $Au_{144}(SC_8H_9)_{60}$ Clusters

Juniper Foxley,[1] Marcus Tofanelli,[2], Jane A. Knappenberger,[1] Christopher J. Ackerson,[2] and Kenneth L. Knappenberger, Jr.[1*]

1. Department of Chemistry, Pennsylvania State University, University Park, PA 16802, USA
2. Department of Chemistry, Colorado State University, Fort Collins, CO 80523, USA

*Corresponding author: klk260@psu.edu

Methods.

$Au_{144}(SC_8H_9)_{60}$ synthesis.

$Au_{144}(PET)_{18}$ was synthesized using the protocol from (ref S1) with slight modifications. In the first step HAuCl4 (0.6 mmol, 236 mg), tetraoctylammonium bromide (TOAB) (0.65 mmol, 380 mg), and 30 ml of methanol were combined in an Erlenmeyer flask, and the solution was stirred for 15 min. Next, 436 µL (3.18 mmol) of phenylethane thiol (PET) was added, immediately generating a white suspension. After 15 min, NaBH4 (6 mmol, 226 mg) was dissolved in 12 ml of ice-cold Nanopure water and added to the gold solution with vigorous stirring for 5 h, at which point the clear supernatant was decanted and discarded, leaving a black, oily mixture. The mixture was washed with methanol, producing a precipitate, which was collected by centrifugation. After washing, the $Au_{25}(PET)_{18}^-$ impurity can be removed by washing with acetonitrile or acetone; however, $Au_{25}(PET)_{18}^0$ will remain. Further purification was performed by size-exclusion chromatography using Bio-Rad SX-1 beads.

Electrochemical characterization.

Electrochemical techniques were performed with a Bioanalytical Systems BAS 100B potentiostat in a DCM solution containing 0.1 M tetrabutylammonium Hexafluorophosphate using a standard calomel electrode as reference.

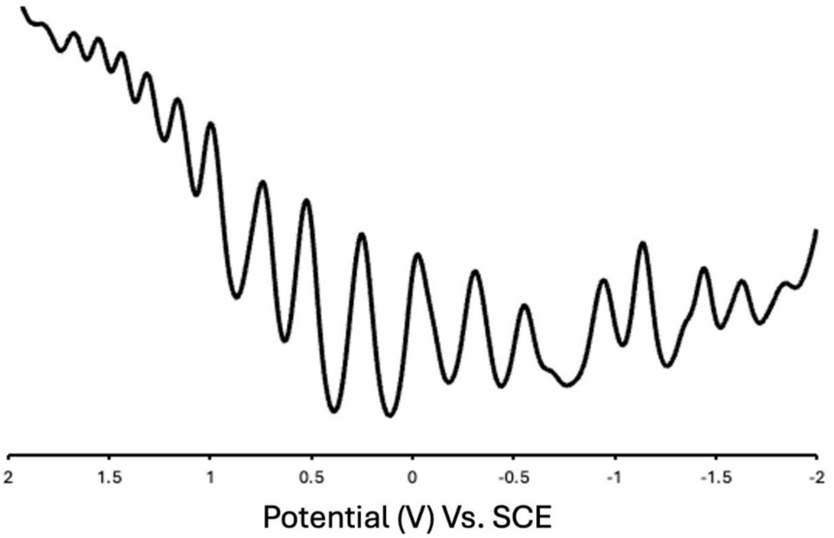

Figure S1. Square Wave Voltammetry of $Au_{144}(SC_8H_9)_{60}$

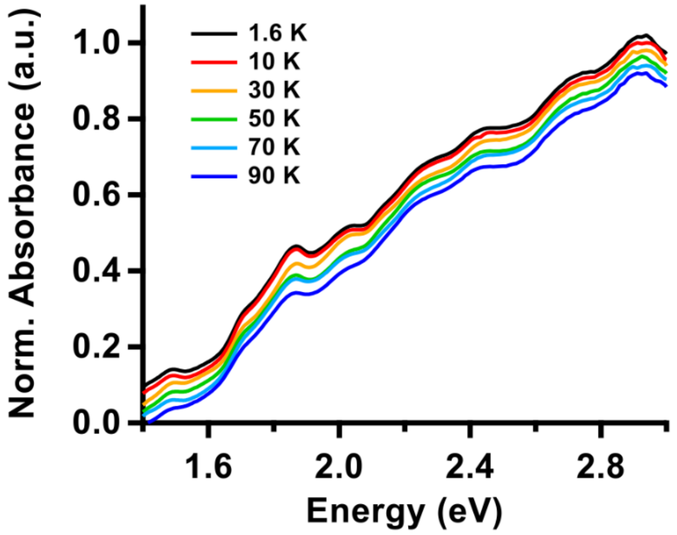

Figure S2. Comparison of $Au_{144}(SC_8H_9)_{60}$ absorption spectra collected at sample temperatures spanning 1.6 K to 90 K. Several distinct absorption features are resolved in the low-temperature absorption spectra.

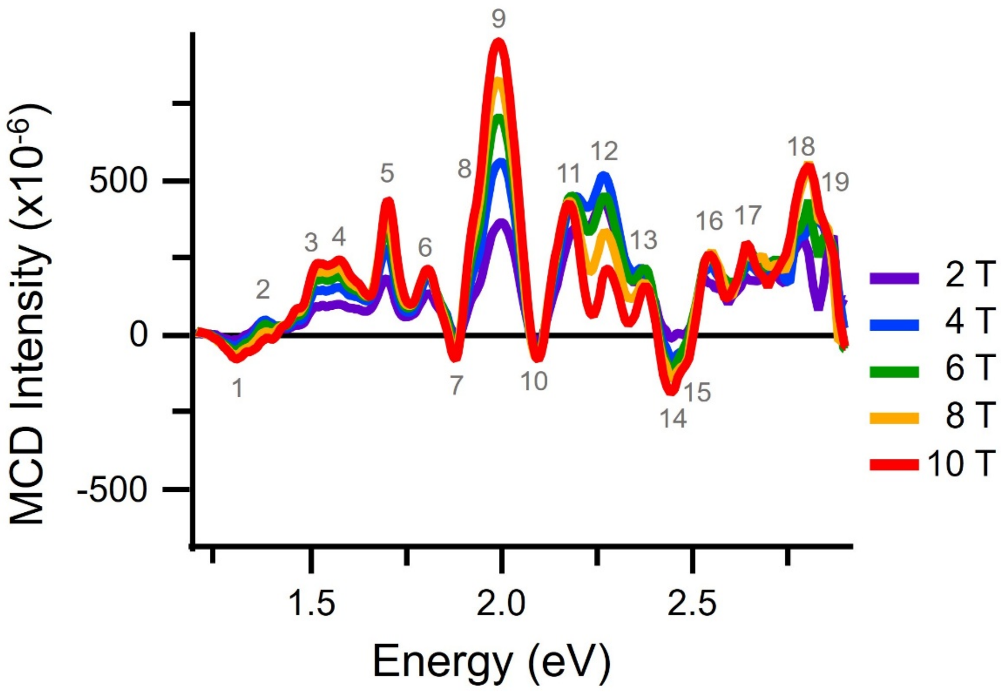

Figure S3. MCD spectra of $Au_{144}(SC_8H_9)_{60}$ at various magnetic field strengths. The 19 observable peaks have been labeled.

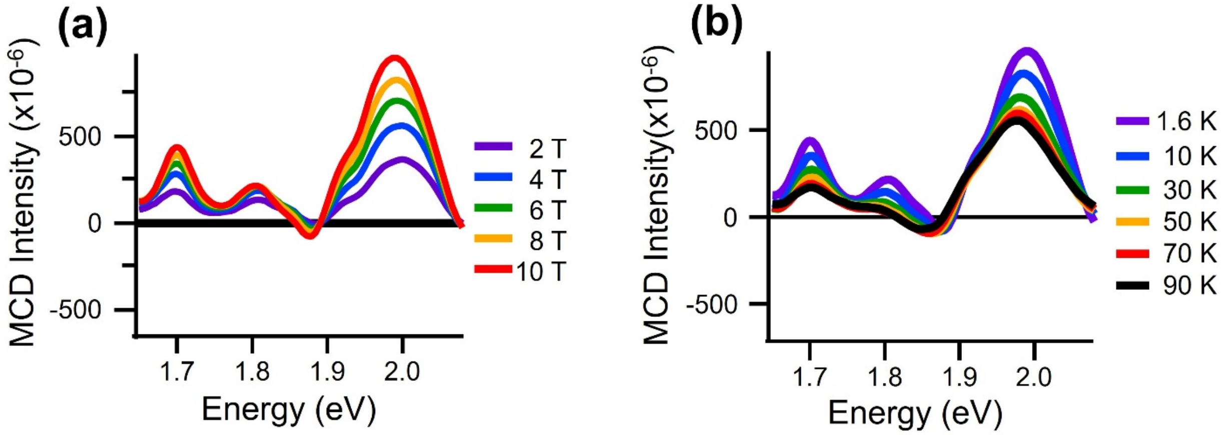

Figure S4. Summary of the MCD response of $Au_{144}(SC_8H_9)_{60}$ truncated to the range between 1.6 eV and 2.1 eV. (a) MCD spectra acquired using applied magnetic field strengths ranging from 2 T to 10 T. Five distinct peaks characterize this region. (b) 10 T MCD spectra acquired at sample temperatures spanning 1.6 K

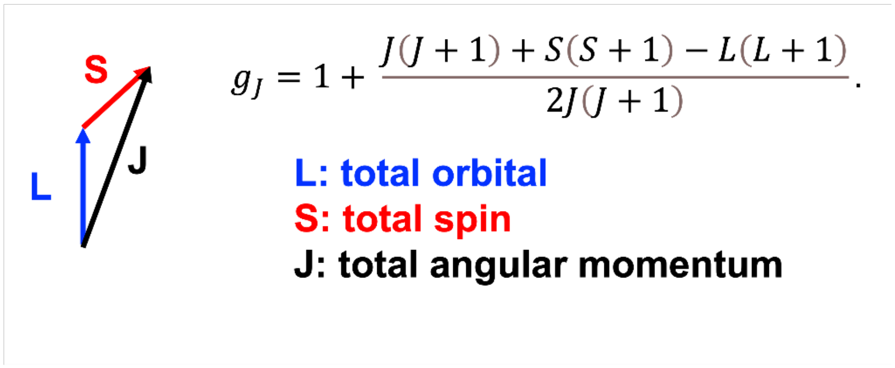

Figure S5. Description of spin-orbit coupling (left) and Lande g-factor (right). The Lande g-factor quantifies the collective interactions from total valence electron orbital angular momentum (L), total spin (S), and the total angular momentum (J).

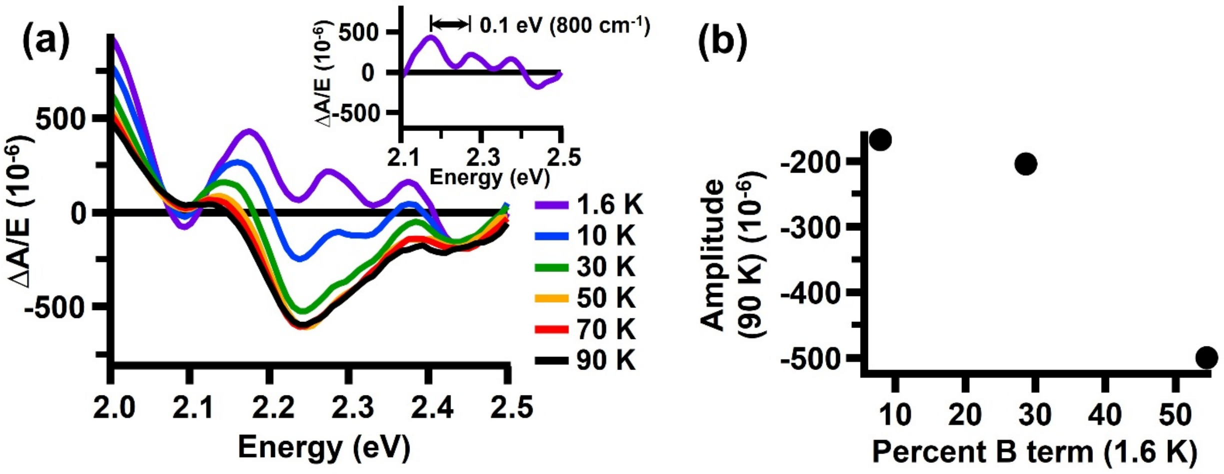

Figure S6. (a) 10 T MCD spectra of $Au_{144}(SC_8H_9)_{60}$ in the range from 2.0 to 2.5 eV acquired for temperatures spanning 1.6 K to 90 K. At temperatures ≥ 10 K, a broad, negative feature dominates the MCD response. At intermediate temperatures, positive-amplitude peaks with periodic spacing are superimposed on the broad, negative peak. The inset shows the regular spacing of these peaks obtained at 10 T and 1.6 K. An average peak-to-peak energy difference of 800 cm^{-1} is observed, consistent with S-C vibrations within monolayer-protected clusters and supporting assignment of these excitations to ligand-band states. (b) The amplitude of the broad, negative component at specified energies as a function of the B-term contribution to each peak for the spectrum collected at 90 K. The observed correlation suggests that the negative-amplitude component underlies the linear magnetization response observed at 1.6 K.

Table S1. All MCD peaks observed for $Au_{144}(SC_8H_9)$. Variable-field MCD analysis discussed in the manuscript focuses on the regions containing peaks 5 through 14.

Peak Number	Peak Energy	MCD Sign	Landé g-factor	% B term
1	1.3	Negative	--	--
2	1.4	Positive	--	--
3	1.55	Positive	--	--
4	1.6	Positive	--	--
5	1.7	Positive	1.9 ± 0.3	30
6	1.8	Positive	1.6 ± 0.3	<10
7	1.85	Negative	2.0 ± 0.8	100
8	1.9	Positive	4.4 ± 1.1	60
9	2.0	Positive	2.1 ± 0.5	45
10	2.1	Negative	--	--
11	2.2	Positive	2.3 ± 0.3	<10
12	2.3	Positive	1.9 ± 0.3	25
13	2.4	Positive	2.1 ± 0.4	20
14	2.45	Negative	--	100
15	2.5	Negative	--	--
16	2.6	Positive	--	--
17	2.7	Positive	--	--
18	2.8	Positive	--	--
19	2.9	Positive	--	--

References

S1. Qian, H.; Jin, R. Chemistry of Materials **2011**, 23, 2209 2217
https://pubs.acs.org/doi/10.1021/cm200143s
