# Supplementary material for: Diverse Superatomic Magnetic and Spin Properties of Au144(SC8H9)60 Clusters
Source: ACS Cent Sci. 2025 May 29;11(8):1329–35. doi: 10.1021/acscentsci.5c00139 (PMC12395290; doi:10.1021/acscentsci.5c00139)
Supplement: Supplementary file 2 [file oc5c00139_si_002.pdf]

oc-2025-001396.R1

Name: Peer Review Information for "Diverse Superatomic Magnetic and Spin Properties of Au144(SC8H9)60 Clusters"

First Round of Reviewer Comments

Reviewer: 1

Comments to the Author

This work is quite informative, and I enjoyed reading.

Briefly, detailed magnetic CD spectroscopic analyses are conducted on the large Au144(SC8H9)60 nanocluster, which approaches the metallic state. Au144 is an important system for understanding the electronic, magnetic and spin properties of quantum-confined metals approaching the classical length scale. This work offers a fundamental understanding of the very complex behavior in Au144. Through variable-field and variable-temperature MCD analyses, two distinct regions are identified, i.e. the 1.5-2 eV region involves superatom orbitals, while the 2-2.5 region involves transitions in the Au-S motifs. Superatomic H orbitals are identified, which may provide new opportunities for exploring spin-dependent chemistry and also quantum information science.

The manuscript is written well. Just a minor thing:

Statement "... lower-energy excitations largely dominated by Au-S-R ligand-centered transitions": but Fig 4 shows that the 1.6-2.0 eV low-energy range is predominantly blue -- i.e. mainly superatomic obital transitions, instead of Au-SR transitions. Pls double check.

Reviewer: 2

Comments to the Author

This manuscript describes a variable temperature and variable magnetic field MCD spectroscopy study of a well-defined Au cluster that exhibits both metallic electronic properties and discrete super-atom like properties with unpaired electrons, giving rise to complex spin-orbit coupling behaviors in the electronic spectra. The broad reach of the study likely comes from the broader interests in such nanoclusters, which bridge nanochemistry and molecular chemistry, for example as catalysts, sensors, or potentially spintronics. The study attempts quantitative analysis of the VTVH MCD spectra, resolving transitions and analyzing them in the context of B/C-terms, to dissect diamagnetic and paramagnetic contributions to the observed transitions. The T/H dependence are interpreted/assigned using previous DFT calculations, connecting the nature of orbitals to the thermally accessible states that the transitions originate from. The study should be of interest to the spectroscopists, and inorganic nanochemists, and those interested in spin-orbit coupled systems, and thus likely suitable for ACS Cent Sci. However, a number of aspects remain unclear, and need to be clarified:

Specific comments:

1. page 3, line 8: what does this term “inorganic diameter” mean? Strange.

2. page 3, line 26-29: “This includes contributions from the superatomic orbitals that originate from Au(I) atoms within the cluster’s core yet are delocalized across the cluster into orbital-like distributions.” Unclear sentence. As stated in earlier text in the intro, Au(I) is in the thirty RS-Au(I)-SR semi-ring units, outside the 114-Au-atom “grand core”. So does the “Cluster’s core” refer to the “grand core” or the entire Au144 cluster?

3. Eq. 1: define “E”. Define “f”.

4. Fig 2a-b: the y-axis quantities should have units. Similarly, Fig 3, 4, 5 should all have units for y-axis.

5. Fig. 2a-b: 19 features were resolved. They should be numbered in the figure, cross-cited in the writing and later figures, to help reading, in correlation with Table S1 of their respective positions in eV and their signs in the MCD spectra.

6. Suggest Table S1 should be placed in main text.

7. A qualitative energy level diagram, depicting Zeeman-splitting with increaseing H, and the multitude of thermal accessible states, would greatly help understand the main message of the paper.

8. Fig. S1: square wave voltammetry: What is the x-axis? No title, no units?

9. Fig. 3: what are the red lines in the figure? Fitting with Eq 2, which is only mentioned for panel d but not for other panels? Please label the panel with energy position and numbering for easier read. Same comments for Fig. 5.

10. page 5, line 53: 6% linear B-term is stated, but it is before the analysis using Eq. (2), which leads to such percentages. Very confusing. Suggest to revise the logic flow.

11. Page 6, line 21: no need to define Bohr magneton and Boltzman constant, which were already defined in Eq 1.

12. Figure 6: how the color was produced, using what data? Specific data presentation procedure should be described. Are the color bars quantitative to some values from the DFT calculation? Such information should be given in the SI.

13. I found the statements like "orbital assignment for this feature would be either 4D3/2 or 4D5/2" very confusing, as 4D3/2 and 4D5/2 are electronic states, not orbitals.

Author's Response to Peer Review Comments:

Kenneth L. Knappenberger, Jr.
Head, Department of Chemistry
Professor of Chemistry and Physics

104 Benkovic Building
University Park, PA 16802

Phone: 814-865-1228
E-mail: klk260@psu.edu

April 20, 2025

Senior Editor, ACS Central Science

Dear Editor:

Uploaded, please find our revised manuscript entitled *"Diverse Superatomic Magnetic and Spin Properties in* $Au_{144}(SC_8H_9)_{60}$ *Clusters"* by Juniper Foxley, Marcus Tofanelli, Jane A. Knappenberger, Christopher Ackerson, and Kenneth L. Knappenberger, Jr. for consideration by *ACS Central Science* as an Article. All co-authors have seen and approved the submission of this manuscript.

We have addressed all comments and questions from both reviewers as well as all editorial requests. Detailed responses to each reviewer comment are attached at the end of this response letter. Uploaded files include one clean manuscript file, and one clean SI file for publication. Our changes to the manuscript can be found highlighted in yellow for ready reference.

We appreciate the thoughtful comments of both reviewers, which we feel have helped us to improve the overall quality of our manuscript. We hope that our responses will adequately address all questions. We look forward to publication of our manuscript in *ACS Central Science*.

Thank you very much for your consideration of our work.

Sincerely,

Kenneth L. Knappenberger, Jr.
Head, Department of Chemistry
Professor of Chemistry
Professor of Physics
Penn State University

Responses to reviewer comments:

Reviewer: 1

Recommendation: Publish in ACS Central Science after minor revisions noted.

Comments:
This work is quite informative, and I enjoyed reading.

We thank the reviewer for this supportive comment.

Briefly, detailed magnetic CD spectroscopic analyses are conducted on the large Au144(SC8H9)60 nanocluster, which approaches the metallic state. Au144 is an important system for understanding the electronic, magnetic and spin properties of quantum-confined metals approaching the classical length scale. This work offers a fundamental understanding of the very complex behavior in Au144. Through variable-field and variable-temperature MCD analyses, two distinct regions are identified, i.e. the 1.5-2 eV region involves superatom orbitals, while the 2-2.5 region involves transitions in the Au-S motifs. Superatomic H orbitals are identified, which may provide new opportunities for exploring spin-dependent chemistry and also quantum information science.

The manuscript is written well. Just a minor thing:
Statement "... lower-energy excitations largely dominated by Au-S-R ligand-centered transitions": but Fig 4 shows that the 1.6-2.0 eV low-energy range is predominantly blue -- i.e. mainly superatomic obital transitions, instead of Au-SR transitions. Pls double check.

We thank the reviewer for this comment. "Au-S-R" has been deleted so as to eliminate any confusion that these are ligand-centered transitions rather than those involving the metal.

Additional Questions:
Quality of experimental data, technical rigor: Top 5%

Significance to chemistry researchers in this and related fields: Top 5%

Broad interest to other researchers: Top 5%

Novelty: Top 5%

Is this research study suitable for media coverage or a First Reactions (a News & Views piece in the journal)?: Yes

Reviewer: 2

Recommendation: Reconsider after major revisions noted.

Comments:
This manuscript describes a variable temperature and variable magnetic field MCD spectroscopy study of a well-defined Au cluster that exhibits both metallic electronic properties and discrete super-atom like properties with unpaired electrons, giving rise to complex spin-orbit coupling behaviors in the electronic spectra. The broad reach of the study likely comes from the broader interests in such nanoclusters, which bridge nanochemistry and molecular chemistry, for example as catalysts, sensors, or potentially spintronics. The study attempts quantitative analysis of the VTVH MCD spectra, resolving transitions and analyzing them in the context of B/C-terms, to dissect diamagnetic and paramagnetic contributions to the observed transitions. The T/H dependence are interpreted/assigned using previous DFT calculations, connecting the nature of orbitals to the thermally accessible states that the transitions originate from. The study should be of interest to the spectroscopists, and inorganic nanochemists, and those interested in spin-orbit coupled systems, and thus likely suitable for ACS Cent Sci. However, a number of aspects remain unclear, and need to be clarified:

We thank the reviewer for this overall, positive assessment.

Specific comments:

1. page 3, line 8: what does this term "inorganic diameter" mean? Strange.

We thank the reviewer for requesting this clarification. The inorganic diameter has now been defined in the revised manuscript.

2. page 3, line 26-29: "This includes contributions from the superatomic orbitals that originate from Au(I) atoms within the cluster's core yet are delocalized across the cluster into orbital-like distributions." Unclear sentence. As stated in earlier text in the intro, Au(I) is in the thirty RS-Au(I)-SR semi-ring units, outside the 114-Au-atom "grand core". So does the "Cluster's core" refer to the "grand core" or the entire Au144 cluster?

We thank the reviewer for bringing this confusion to our attention. We have revised this text to clarify. We have edited this text to read as follows: This includes contributions from the superatomic orbitals that originate from Au(0) atoms within the cluster's core.

3. Eq. 1: define "E". Define "f".

We thank the reviewer for noting that these terms were not defined. E and f have been defined in the revision.

4. Fig 2a-b: the y-axis quantities should have units. Similarly, Fig 3, 4, 5 should all have units for y-axis.

The y-axis for all referenced figures is now labeld as "MCD intensity," a term which has been defined in the text as the difference spectra (left-right) of left and right circularly polarized absorption.

5. Fig. 2a-b: 19 features were resolved. They should be numbered in the figure, cross-cited in the writing and later figures, to help reading, in correlation with Table S1 of their respective positions in eV and their signs in the MCD spectra.

We thank the reviewer for this suggestion. A figure containing labels for all peaks has been added to the SI. The figure used in the main manuscript has ranges of peaks labeled. Throughout the manuscript, peaks have been identified both by number and by energy. We believe this change will help improve the readability of our manuscript.

6. Suggest Table S1 should be placed in main text.

We thank the reviewer for this suggestion. A table describing the peaks discussed in the manuscript has been added to the main manuscript. A full table describing all 19 peaks is now located in the SI. These tables contain peak number, MCD sign, Lande g factor, and approximate %B. The table in the SI also contains the sign of the MCD signal.

7. A qualitative energy level diagram, depicting Zeeman-splitting with increaseing H, and the multitude of thermal accessible states, would greatly help understand the main message of the paper.

An energy diagram showing Zeeman splitting has now been added to the revised manuscript as Figure 2.

8. Fig. S1: square wave voltammetry: What is the x-axis? No title, no units?

The x-axis of Figure S1 has now been appropriately labeled.

9. Fig. 3: what are the red lines in the figure? Fitting with Eq 2, which is only mentioned for panel d but not for other panels? Please label the panel with energy position and numbering for easier read. Same comments for Fig. 5.

We thank the reviewer for this suggestion. The red lines have been described in the Figure caption. Individual panels are now labeled with peak number and energy.

10. page 5, line 53: 6% linear B-term is stated, but it is before the analysis using Eq. (2), which leads to such percentages. Very confusing. Suggest to revise the logic flow.

We thank the reviewer for this suggestion. The discussion of approximate %B values has now been moved to a more appropriate location, found after equation 2.

11. Page 6, line 21: no need to define Bohr magneton and Boltzman constant, which were already defined in Eq 1.

The superfluous definitions have been removed.

12. Figure 6: how the color was produced, using what data? Specific data presentation procedure should be described. Are the color bars quantitative to some values from the DFT calculation? Such information should be given in the SI.

We thank the reviewer for requesting clarification on this. A sentence noting that color is derived from relative contributions noted in previous DFT calculations has been added to the manuscript. We think this revision helps increase understanding of the work.

13. I found the statements like "orbital assignment for this feature would be either 4D3/2 or 4D5/2" very confusing, as 4D3/2 and 4D5/2 are electronic states, not orbitals.

We thank the reviewer for pointing this out. The word "orbital" has been removed to avoid confusion.

Additional Questions:
Quality of experimental data, technical rigor: High

Significance to chemistry researchers in this and related fields: High

Broad interest to other researchers: Moderate

Novelty: Moderate

Is this research study suitable for media coverage or a First Reactions (a News & Views piece in the journal)?: No

oc-2025-001396.R2

Name: Peer Review Information for "Diverse Superatomic Magnetic and Spin Properties of Au144(SC8H9)60 Clusters"

Second Round of Reviewer Comments

Reviewer: 1

Comments to the Author

The revised manu is ready for publication.

Reviewer: 2

Comments to the Author

The authors have addressed my comments sufficiently. Publication recommended.

Author's Response to Peer Review Comments:

We thank the reviewers for their positive evaluation of our manuscript. We have uploaded a revised copy with the requested synopsis. We look forward to publication of our manuscript in ACS Central Science.

Best regards,

Kenneth L. Knappenberger, Jr.
